# Inhibition of DNA Repair Pathways and Induction of ROS Are Potential Mechanisms of Action of the Small Molecule Inhibitor BOLD-100 in Breast Cancer

**DOI:** 10.3390/cancers12092647

**Published:** 2020-09-16

**Authors:** Suzanne Bakewell, Isabel Conde, Yassi Fallah, Mathew McCoy, Lu Jin, Ayesha N. Shajahan-Haq

**Affiliations:** 1 Preclinical Development, Intezyne Technologies, Tampa, FL 33612, USA; suzanne.bakewell@libertybiosecurity.com; 2 Department of Oncology, Lombardi Comprehensive Cancer Center, Georgetown University Medical Center, Washington, DC 20057, USA; ic364@georgetown.edu (I.C.); yf120@georgetown.edu (Y.F.); mdm299@georgetown.edu (M.M.); lj74@georgetown.edu (L.J.); 3 Innovation Center for Biomedical Informatics (ICBI), Georgetown University Medical Center, Washington, DC 20057, USA

**Keywords:** breast cancer, BOLD-100, olaparib, triple negative breast cancer

## Abstract

**Simple Summary:**

BOLD-100 is a novel ruthenium-based drug that has shown anti-tumor effects in several human cancers. In this study, we investigated the biochemical changes associated with BOLD-100 in human breast cancer cell models to better understand how this drug inhibits cancer cell growth. We found that treatment with BOLD-100 induced chemically unstable reactive oxygen species (ROS) and reduced proteins that help repair DNA in breast cancer cells. When combined with other drugs that target the DNA repair pathway, such as olaparib, BOLD-100 promoted more DNA damage and cell death compared with olaparib alone in estrogen receptor negative (ER-) breast cancer cells. ER- breast cancer is an aggressive form of breast cancer without any specific targeted therapy. Thus, our findings provide the rationale for a novel combination therapeutic strategy with BOLD-100 for ER- breast cancer subtype that could be investigated in clinical trials.

**Abstract:**

BOLD-100, a ruthenium-based complex, sodium trans-[tetrachloridobis (1H-indazole) ruthenate (III)] (also known as IT-139, NKP1339 or KP1339), is a novel small molecule drug that demonstrated a manageable safety profile at the maximum tolerated dose and modest antitumor activity in a phase I clinical trial. BOLD-100 has been reported to inhibit the upregulation of the endoplasmic reticulum stress sensing protein GRP78. However, response to BOLD-100 varies in different cancer models and the precise mechanism of action in high-response versus low-response cancer cells remains unclear. In vitro studies have indicated that BOLD-100 induces cytostatic rather than cytotoxic effects as a monotherapy. To understand BOLD-100-mediated signaling mechanism in breast cancer cells, we used estrogen receptor positive (ER+) MCF7 breast cancer cells to obtain gene-metabolite integrated models. At 100 μM, BOLD-100 significantly reduced cell proliferation and expression of genes involved in the DNA repair pathway. BOLD-100 also induced reactive oxygen species (ROS) and phosphorylation of histone H2AX, gamma-H2AX (Ser139), suggesting disruption of proper DNA surveillance. In estrogen receptor negative (ER−) breast cancer cells, combination of BOLD-100 with a PARP inhibitor, olaparib, induced significant inhibition of cell growth and xenografts and increased gamma-H2AX. Thus, BOLD-100 is a novel DNA repair pathway targeting agent and can be used with other chemotherapies in ER− breast cancer.

## 1. Introduction

In the United States, about 150,000 patients are currently living with metastatic breast cancer. While targeted therapies have improved survival, metastatic breast cancer remains an incurable disease. Therefore, there is an urgent need for developing therapies that could provide novel treatment options for such patients. Breast cancer is a heterogeneous disease that is broadly classified into hormone-receptor-positive, estrogen receptor positive and progesterone receptor positive (ER+/PR+), human epidermal growth factor receptor-2 overexpressing (HER2+) or triple-negative breast cancer (TNBC or ER−; lacks ER, PR and HER2). Seventy percent of all breast cancers are ER+/PR+ and are treated with endocrine therapy that blocks ER activity with antiestrogens such as Tamoxifen or Faslodex/Fulvustrant/ICI or aromatase inhibitors such as letrozole [1,2]. Advanced ER+/PR+ disease is currently treated with endocrine therapy and CDK4/6 inhibitors [3,4,5]. HER2 is overexpressed in 15–20% of breast cancers and treatment is targeted with anti-HER2 targeting agents [6]. TNBC constitutes about 10–15% of all breast cancers [7], and although BRCA-associated TNBC is treated with inhibitors of the enzyme poly ADP ribose polymerase (PARP; e.g., olaparib), this subtype remains without a defined drug target and is treated with chemotherapies [8]. In addition to these molecular classifications, other factors such as menopausal status, lymph node invasion and tumor size are considered to determine the best therapeutic option. However, the ability of cancer cells to evade cell death and to develop drug resistance remains a major clinical hurdle [9,10,11]. Therefore, novel therapies that are effective in inducing cell death in combination with standard of care therapies are urgently needed to improve survival in breast cancer patients.

BOLD-100 is an intravenously administered small molecule shown to be well tolerated with modest anti-tumor activity in a Phase I clinical trial [12]. A high affinity for albumin and unique chemistry allow increased activation of BOLD-100 within tumors cells. BOLD-100 can bind non-covalently with albumin through hydrophobic interactions and can be activated within cells upon release from albumin [13,14]. BOLD-100′s mechanism has recently shown to be partly due to the suppression of GRP78 at the mRNA and protein level in thapsigargin induced stressed or drug resistant cancer cells in a context-dependent manner [15,16,17]. In this study, we treated non-stressed breast cancer cells with BOLD-100 and established an integrated signaling network to look at significantly altered genes and metabolites. This integrated signaling network showed that BOLD-100 altered DNA repair pathways. Both in vitro and in vivo studies show that BOLD-100 is a useful combination strategy with other DNA targeting drugs, particularly, the PARP-inhibitor olaparib in TNBC. Thus, BOLD-100 may be beneficial in combination with other treatment strategies that target DNA repair pathways in cancer. 

## 2. Results

### 2.1. BOLD-100 Inhibits Growth of Breast Cancer Cells

**Figure 1:**
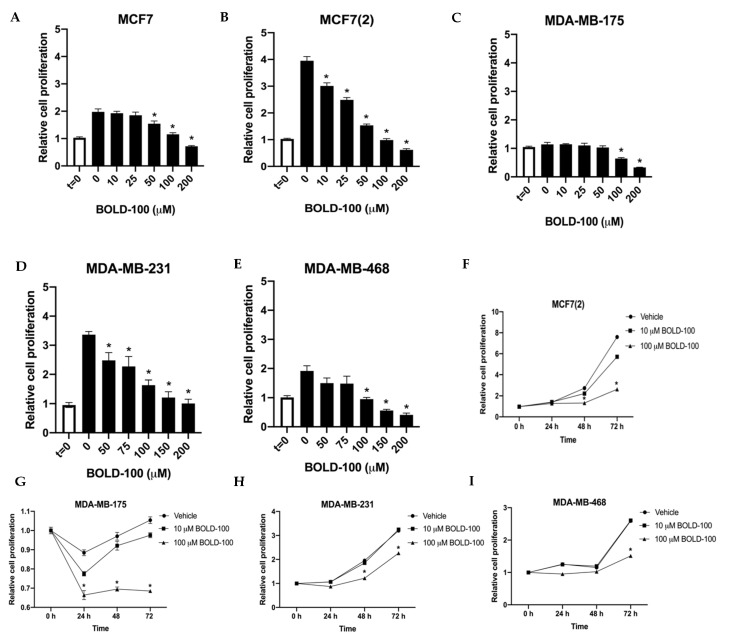
BOLD-100 inhibited growth of breast cancer cells. Estrogen receptor positive (ER+) breast cancer cells [MCF7, MCF7(2) and MDA-MB-175] and estrogen receptor negative (ER−) breast cancer cells (MDA-MB-231 and MDA-MB-468) were treated with indicated concentrations of BOLD-100 for 72 h and compared to time = 0 (at the onset of treatment). (**A**) MCF7(2), (**B**) MCF7, (**C**) MDA-MB-175, (**D**) MDA-MB-231 and (**E**) MDA-MB-468 cells were significantly (* *p* < 0.05) inhibited by 100 μM BOLD-100. Time-course (24, 48 and 72 h) with 0, 10 or 100 μM BOLD-100 showed significant (* *p* < 0.04) inhibition of (**F**) MCF7(2), (**G**) MDA-MB-175, (**H**) MDA-MB-231 and (**I**) MDA-MB-468 cells with 100 μM compared with 0 μM BOLD-100 at 72 h. ANOVA, *p* < 0.01; * *p* < 0.05 for conditions in indicated cells compared with control.

**Figure 2:**
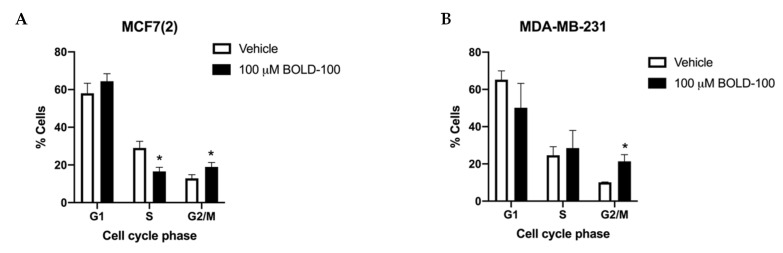
BOLD-100 treatment arrested breast cancer cells in G2/M phase of the cell cycle. Cell cycle analysis of (**A**) MCF7(2) and (**B**) MDA-MB-231 cells with 100 μM of BOLD-100 for 72 h showed significant decrease in S phase and increase in G2/M compared with vehicle treated cells. ANOVA, *p* < 0.01; * *p* < 0.05 for respective cell cycle phase in cells treated with 100 μM BOLD-100 compared with cells treated with vehicle.

To determine the effect of BOLD-100 in non-stressed breast cancer cells, we treated both ER+ [MCF7, MCF7(2), MDA-MB-175] and TNBC [MDA-MB-231, MDA-MB468] breast cancer cells in regular cell culture media under basal conditions with BOLD-100 ranging from 10 to 200 μM for 72 h (Figure 1A–E). MCF7(2) is a MCF7 derivative cell line that shows increased sensitivity to estrogen [18]. Since cell growth rates vary in the different cell lines, we used t = 0 to compare the net growth at 72 h with vehicle (DMSO) alone or different doses of BOLD-100. All cells showed varying levels of sensitivity to BOLD-100 at different doses. For all cells, growth was significantly (*p* < 0.05) inhibited with 100 μM BOLD-100 compared with vehicle at 72 h. Particularly, MCF7(2), MDA-MB-231 and MDA-MB-468 cells (IC50 ~100 μM) showed increased sensitivity to BOLD-100 in a dose-dependent manner. Time-course studies with 0, 10 or 100 μM BOLD-100 showed significant (*p* < 0.04) decrease in cell proliferation with 100 μM compared with control conditions at 72 h in MCF7(2), MDA-MB-175, MDA-MB-231 and MDA-MB-468 cells (Figure 1F–I). Analysis of cell cycle in MCF7(2) and MDA-MB-231 cells, two cell lines that showed increased sensitivity (*p* < 0.05), showed significant increase in cells in G2/M phase treated with 100 μM BOLD-100 compared with vehicle (Figure 2A,B). Therefore, BOLD-100 mediated inhibition of cell proliferation involves G2/M cell cycle arrest.

### 2.2. Significantly Altered Genes, Metabolites and Proteins in BOLD-100 Treated Cells Show Changes in DNA Repair Pathways

**Figure 3:**
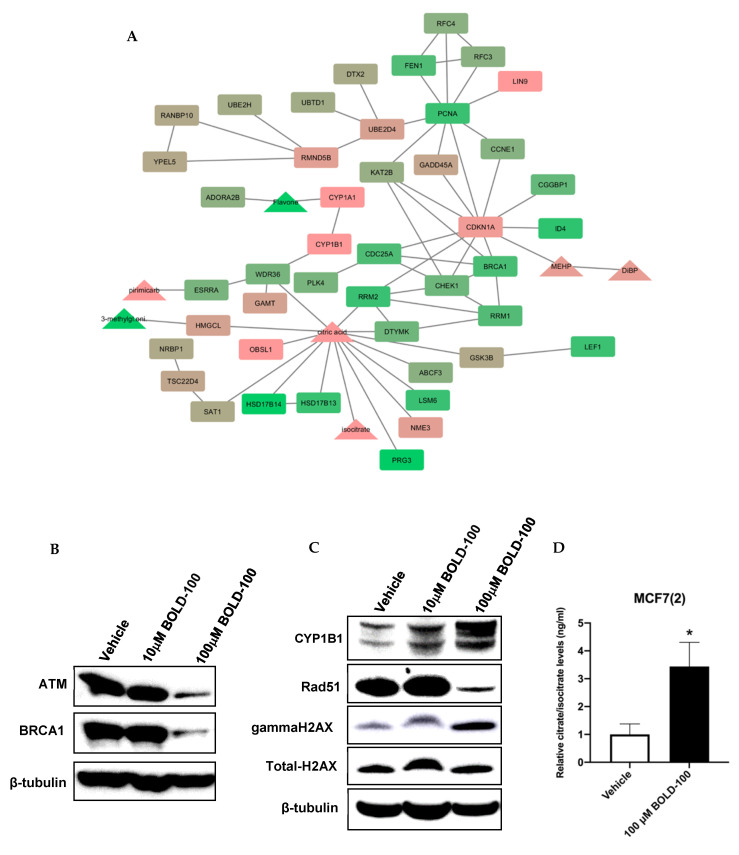
Integration and validation of significantly altered proteins in MCF7(2) cells treated with 100 μM BOLD-100 for 72 h. (**A**) Network analysis of the top 2000 differentially expressed (DE) genes, ranked by *p*-value. Each of the mutually exclusive networks of DE genes were input into STITCH along with all DE metabolites to integrate the gene and protein expression datasets and expand the networks to include common interacting neighbors from the STITCH database. The resulting networks for each DE set were integrated and pruned of both nodes not detected as differentially expressed in the dataset including subnetworks isolated from the largest interconnected sets. The networks were visualized using Cytoscape, with genes represented as rectangles and metabolites as triangles, with the log2 fold change overlaid scaled from low (green) to high (red) expression. (**B**–**D**), Western blot analysis in cells treated with vehicle (DMSO), 10 μM or 100 μM BOLD-100 for 72 h show (**B**) ATM and BRCA1 protein levels were decreased with 100 μM BOLD-100 treatment, (**C**) CYP1B1 and gamma-H2AX (marker of DNA double-strand break) was increased (total H2AX levels did not change) while RAD51 protein level was decreased with 100 μM BOLD-100 treatment. For whole blots, please see Figures S3 and S4. (**D**) Mass spectrometry based quantitative measurement show significant (* *p* < 0.01, Student *t*-test) increase in citric acid and isocitrate following treatment with 100 μM BOLD-100 in MCF7 cells.

Previously, it has been shown that BOLD-100 inhibits the upregulation of GRP78 in stressed cancer cells [15]. To determine the molecular changes associated with BOLD-100 treatment in non-stressed cells, we analyzed and integrated the signaling pathways associated with significantly changed genes and metabolites in MCF7(2) cells (Figure 3A). We selected MCF7(2) cells for molecular analysis based on their high sensitivity to BOLD-100 as demonstrated by cell growth studies shown above (Figure 1). MCF7(2) cells were treated with vehicle alone or 100 μM BOLD-100 for 72 h followed by transcriptomics and metabolomics analysis (see Section 4.6 for details). Network analysis of the top 2000 differentially expressed genes, ranked by *p*-value, [IPA Core Analysis (QIAGEN Inc., Redwood City, CA, USA)] identified disease and functional pathways enriched in differentially expressed genes (Table S1). Networks of differentially expressed genes were input into STITCH [19] along with differentially expressed metabolites, to integrate the gene expression datasets and expand the networks to include common interacting neighbors from the STITCH database. The resulting networks for each differentially expressed set were integrated and pruned of both nodes not detected as differentially expressed in the dataset including subnetworks isolated from the largest interconnected sets. The networks were visualized using Cytoscape [20] with genes represented as rectangles and metabolites as triangles, with the log2-fold change overlaid scaled from low (green) to high (red) expression (Figure 3A). Gene expression levels for GRP78 was not changed in treated versus control cells in this analysis, which was further validated by assessing GRP78 protein levels with Western blot analysis (Figures S1A and S7). Differentially exprsed genes-metabolites network comprised several genes associated with DNA damage response that were significantly decreased, such as ATM, CHEK1, BRCA1, CDC25A while cell cycle arrest genes were significantly increased, such as CDKN1A(p21) and LIN9 (Figure 3A). Selected proteins coded by the genes in the network was validated by Western blot analysis in MCF7(2) cells treated with 0, 10 or 100 μM BOLD-100 for 72 h to show the dose-dependent effect of the drug on protein levels (Figure 3B,C, Figures S3 and S4). Interestingly, CYP1B1, a metabolizing enzyme involved in processing of xenobiotics or drugs [21] was increased at both the gene and protein levels (Figure 3A,C). Furthermore, mass spectrometry based quantitative measurement of citric acid and isocitrate, critical intermediates of the Tricarboxylic Acid Cycle (TCA cycle), showed 11-fold elevated in BOLD-100 treated cells compared to control (Figure 3D). Furthermore, high throughput analysis of proteins using Reverse Phase Protein Array (RPPA) [22] in MCF7(2) cells treated with vehicle or 100 μM BOLD-100 for 72 h showed significant decrease in cell cycle proteins such as RAD51, PCNA, ATM and ATRX (Table 1; for a complete list of all significantly changed proteins with BOLD-100 treatment see Table S2), consistent with the gene expression analysis.

### 2.3. BOLD-100 Induces Reactive Oxygen Species (ROS)

**Figure 4:**
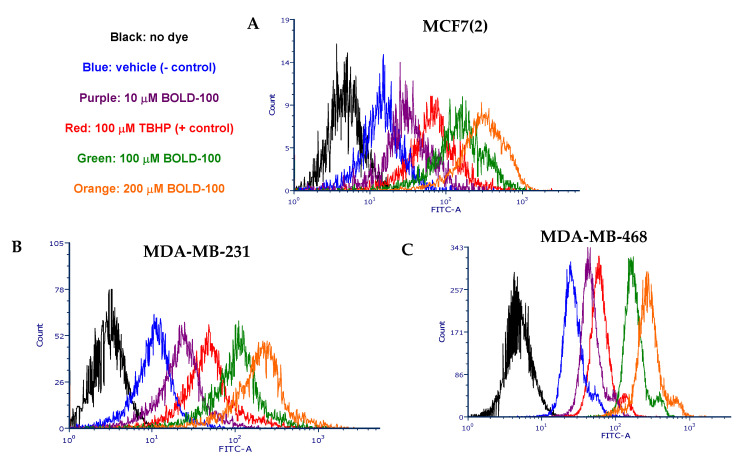
BOLD-100 induced ROS in breast cancer cells. Cell permeant reagent 2′,7′ –dichlorofluorescin diacetate (DCFDA) was used to measure cellular ROS activity (FITC-A, x-axis) in within cells (count, y-axis). BOLD-100 induced ROS increased in a dose-dependent manner in (**A**) MCF7(2), (**B**) MDA-MB-231 and (**C**) MDA-MB-468 cells. Negative control was the cells treated with vehicle (DMSO alone) and positive control was the cells treated with 100 μM Tert-Butyl Hydrogen Peroxide (TBHP) for 6 h.

Since DNA repair pathway genes were significantly increased in BOLD-100 treated cells, we compared total cellular ROS in untreated and treated cells. ROS can induce DNA damage and trigger a complex signaling mechanism called the DNA damage response (DDR) that is used by the cell to reset genomic stability [23]. In MCF7(2), MDA-MB-231 and MDA-MB-468 cells, treatment with BOLD-100 increased ROS levels in a dose-dependent manner (Figure 4A–C) compared with vehicle treated cells (negative control). In fact, at 100 and 200 μM, BOLD-100 induced more ROS than 100 μM tert-butyl hydroperoxide (TBHP), which is a positive control for inducing cellular ROS [24]. Thus, these data suggest that the anti-proliferative effects of BOLD-100 in breast cancer cells is partly due to ROS-dependent cellular damage.

### 2.4. BOLD-100 Synergizes with Anticancer Agents that Target DNA in TNBC Cells

**Figure 5:**
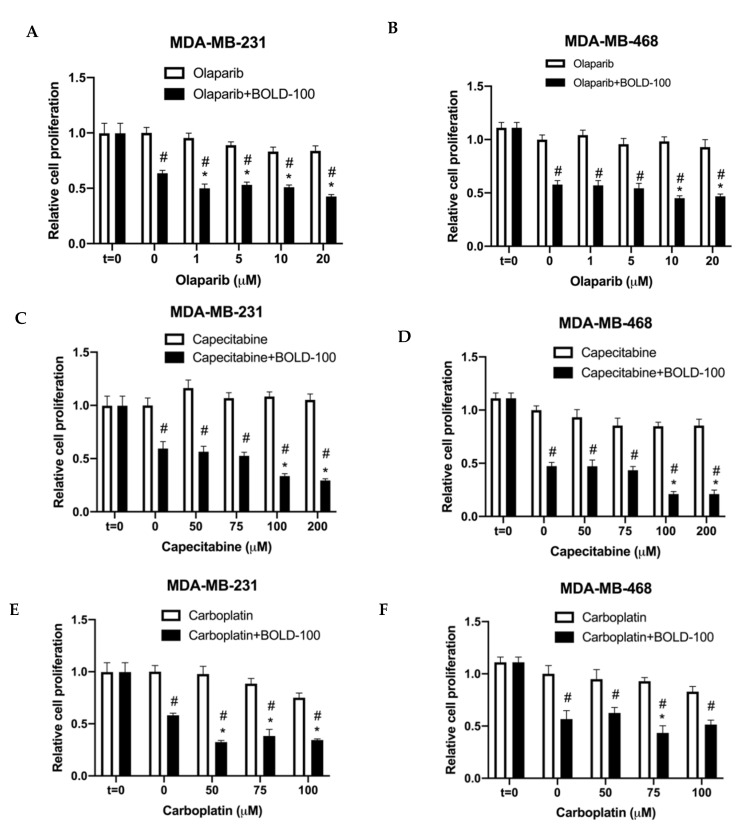
BOLD-100 suppressed growth of TNBC breast cancer cells in combination with anticancer drugs that target the DNA. Cell proliferation was measured using a crystal violet method. Synergistic interactions were analyzed by calculating RI values (see Section 4.11; RI >1 indicates synergy). (**A**) MDA-MB-231 and (**B**) MDA-MB-468 cells were treated with varying concentrations of olaparib and 100 μM BOLD-100. (**C**) MDA-MB-231 and (**D**) MDA-MB-468 cells were treated with varying concentrations of capecitabine and 100 μM BOLD-100. (**E**) MDA-MB-231 and (**F**) MDA-MB-468 cells were treated with varying concentrations of caboplatin and 100 μM BOLD-100. ANOVA, *p* < 0.001; # *p* < 0.05 for treatment versus control for respective cell lines; * *p* < 0.05 for treatment versus 0 μM for specific drug in respective cell lines.

Drugs that target DNA synthesis or repair pathways are often used to treat TNBC. For example, olaparib, an inhibitor of PARP; capecitabine, an oral antimetabolite that interferes with DNA synthesis; or carboplatin, a DNA damaging agent [25]. Hence, we tested the efficacy of BOLD-100 in combination with these DNA targeting agents in TNBC cells. MDA-MB-231 and MDA-MB-468 cells were treated with increasing doses of olaparib, capecitabine or carboplatin in combination with 100 μM BOLD-100. For all conditions, t = 0 was included to compare growth rates of cells under different conditions from the onset of treatments. Synergistic interactions were analyzed by calculating RI values (see Section 4.11; RI > 1 indicates synergy) [26]. In MDA-MB-231 cells, combination of BOLD-100 and olaparib synergistically inhibited cell proliferation at all concentrations from 1 μM (R = 1.21), 5 μM (R = 1.06), 10 μM (R = 1.04) and 20 μM (R = 1.25) olaparib compared with BOLD-100 alone. In MDA-MB-468 cells, combination of BOLD-100 and olaparib synergistically inhibited cell proliferation at 10 μM (RI = 1.26) and 20 μM (R = 1.14) (Figure 5A,B). For capecitabine, 100 μM BOLD-100 synergized with 100 μM (R = 1.91) and 200 μM (R = 1.92) of the drug (Figure 5C,D) in both MDA-MB-231 and MDA-MB-468 cells. For carboplatin, BOLD-100 synergized with 50 μM (R = 1.75), 75 μM (R = 1.34) and 100 μM (R = 1.26) carboplatin in MDA-MB-231 cells but only with 75 μM carboplatin in MDA-MB-468 cells (Figure 5E,F).

**Figure 6:**
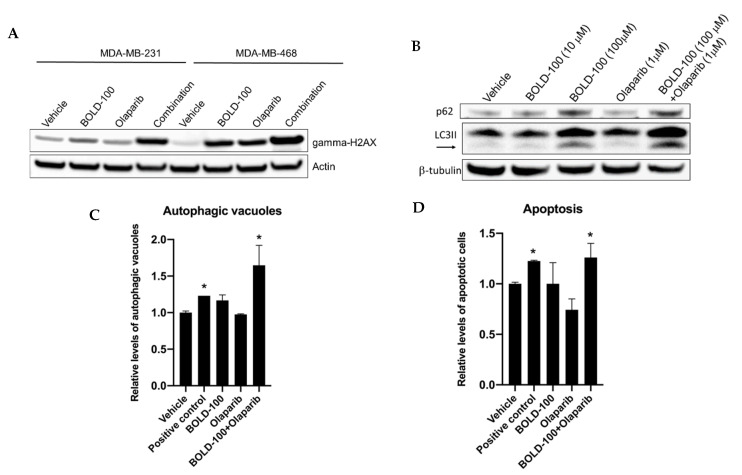
Combination of BOLD-100 and olaparib induces γH2AX and cell death in TNBC cells. (**A**) Increased gamma-H2AX protein levels were detected by Western blotting in MDA-MB-231 and MDA-MB-468 cells following treatment with both BOLD-100 and olaparib compared with vehicle control for 72 h. Actin was used as a loading control. (**B**) Western blot analysis in MDA-MB-231 cells treated with BOLD-100 (100 μM) or BOLD-100 (100 μM) + olaparib (1 μM) showed increased LC3II (autophagosome marker) and p62 (marker of autophagy activity). β-tubulin was used as a loading control. For whole blots, see Figures S5 and S6. (**C**) Cyto-ID assay (Enzo, Farmingdale, NY, USA) was used to measure autophagic vacuoles in MDA-MB-231 cells using flow cytometry: vehicle alone, positive control (cells were serum deprived for 24 h), 100 μM BOLD-100, olaparib (1 μM) or the combination at 72 h. Significantly increased autophagic vacuoles were seen in cells treated with both BOLD-100 and olaparib. ANOVA, *p* < 0.001; * *p* < 0.001 for treatment versus vehicle. (**D**) Annexin V-FITC assay was used to measure apoptosis levels in MDA-MB-231 cells: vehicle alone, positive control (10 nM doxorubicin), 100 μM BOLD-100, olaparib (1 μM) or the combination at 72 h. ANOVA, *p* < 0.002; * *p* < 0.03 for treatment versus vehicle.

To further elucidate how BOLD-100 increases efficacy of DNA targeting anticancer drugs, we focused on the combination of olaparib and BOLD-100. In cancer cells, ROS has been implicated in mediating therapeutic response [27] by inducing DNA damage. Particularly, double stranded breaks (DSB) in DNA is commonly detected with phosphorylation (S139) of the histone H2AX (gamma-H2AX) [28,29]. Since we showed that BOLD-100 induces ROS (Figure 4), to assess whether BOLD-100 induced DSB, we analyzed gamma-H2AX protein in TNBC cells treated with BOLD-100 and/or olaparib (Figure 6A and Figure S5). When treated with both olaparib (10 μM) and BOLD-100 (100 μM), increased levels of gamma-H2AX were observed in both MDA-MB-231 and MDA-MB-468 cells relative to treatments with individual drugs or vehicle alone. While treatment with BOLD-100 or olaparib alone did not increase gamma-H2AX protein levels in MDA-MB-231 cells, these drugs increased gamma-H2AX levels in MDA-MB-468 cells as single agents compared with vehicle. However, in both cells, gamma-H2AX levels were most pronounced with combination of both drugs.

To better understand the mode of cell death pathway following this combination treatment, we evaluated levels of autophagy and apoptosis (Figure 6B–D). MDA-MB-231 cells treated with Bold-100 alone or in combination with olaparib increased accumulation of autophagosome marker, LC3II, and autophagosome cargo protein, p62 (SQSTM1) (Figure 6B and Figure S6). Both LC3II and p62 were increased, which implies a blockage of autophagy [30]. Measurement of autophagosomes using CytoID dye that accumulates within autophagosomes showed significantly (*p* < 0.001) increased levels of these vacuoles in cells treated with olaparib and Bold-100 compared with vehicle alone (Figure 6C). Cells that were serum starved for 24 h was used as positive control. Furthermore, measurement of apoptotic cells using annexin-V in MDA-MB-231 cells showed significantly more cells undergoing apoptosis when treated with olaparib and Bold-100 compared to vehicle treated cells. Doxorubicin (10 nM) was used as a positive control to induce apoptosis. Together, these data suggest that combination of olaparib and BOLD-100 blocked autophagic flux and induced cell death via apoptosis.

### 2.5. Combination of BOLD-100 and Olaparib Suppressed Growth of TNBC Tumors

**Figure 7:**
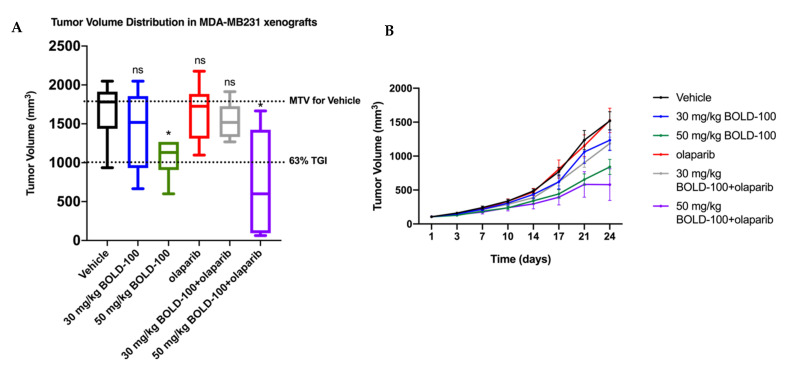
Combination of BOLD-100 and olaparib suppressed growth of TNBC xenografts. (**A**) Box and whisker plot showing distribution of tumor volume in each group of nude mice with MDA-MB-231 xenografts on Day 24 after treatment. Tumors treated with the combination of 50 mg/kg BOLD-100 and olaparib showed a 63% tumor growth inhibition (TGI), (TGI > 60% suggest a potential therapeutic activity), that differed significantly (* *p* ≤ 0.05, Mann–Whitney test) from vehicle or olaparib monotherapy but was not significantly different from BOLD-100 50 mg/kg. ns, not significant. (**B**) The mean tumor volumes in each treatment group were plotted as a function of time (days). For both graphs, colors are: vehicle (black), 30 mg/kg BOLD-100 (blue), 50 mg/kg BOLD-100 (green), olaparib (red), 30 mg/kg BOLD-100+olaparib (grey) and 50 mg/kg BOLD-100+olaparib (purple).

**Figure 8:**
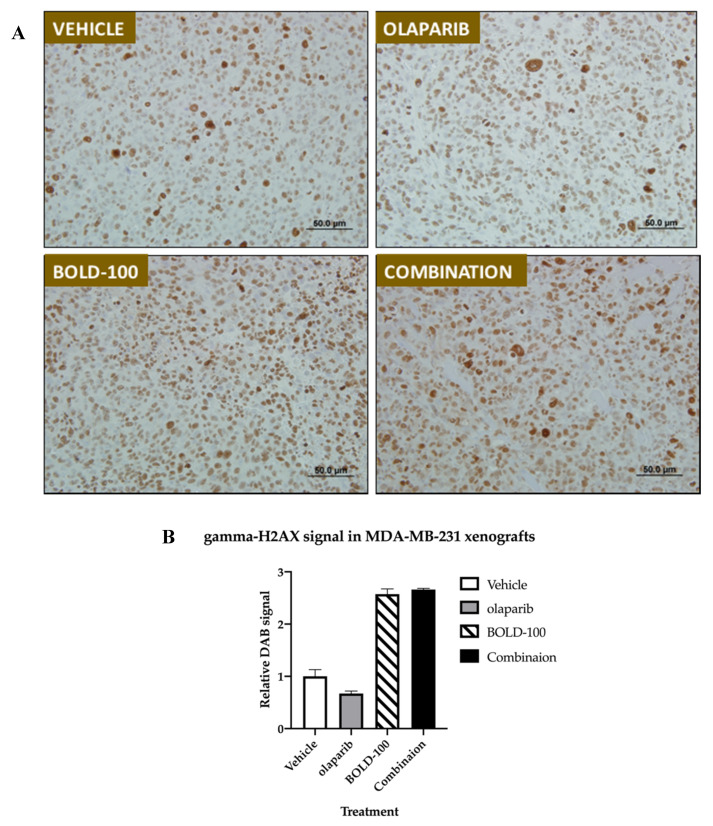
Treatment with BOLD-100 or BOLD-100+olaparib increased gamma-H2AX protein levels in MDA-MB-231 xenografts. **(A)** Immunohistochemical (IHC) gamma-H2AX staining show increased protein levels (diaminobenzidine (DAB), brown) in tumors treated with BOLD-100 (50 mg/kg) or BOLD-100 (50 mg/kg)+olaparib compared with vehicle or olaparib only treated tumors. (**B**) Semi-quantitative analysis of DAB signal (see Section 4.9) shows increased gamma-H2AX in tumors treated with BOLD-100 compared with control.

To further validate the efficacy of BOLD-100 and olaparib in vivo, we tested this combination in MDA-MB-231 breast cancer xenografts (Figure 7). Female nude mice with established subcutaneous MDA-MB-231 tumors were treated in accordance with the protocol summarized in Table 2 in six groups. Distribution of tumor volume in each group on Day 24 is shown in box and whisker plots (Figure 7A). Group 1 received saline, and served as the control group for analysis of tumor growth inhibition (TGI) and as a standard of statistical comparison for all treatment groups. Two different BOLD-100 doses were followed: Group 2 mice received BOLD-100 at 30 mg/kg while Group 3 animals received BOLD-100 at 50 mg/kg. Group 4 animals received olaparib at 50 mg/kg. Group 5 animals received BOLD-100 at 30 mg/kg in combination with olaparib. Group 6 animals received BOLD-100 at 50 mg/kg in combination with olaparib. Mean tumor volume (MTV) in Group 3 that received 50 mg/kg BOLD-100 as a monotherapy showed 45% tumor growth inhibition (TGI) that differed significantly (*p* < 0.05) from the control Group 1. Tumors treated with the combination of 50 mg/kg BOLD-100 and olaparib (Group 6) showed a 63% TGI that differed significantly (*p* < 0.05) from the control Group 1 and olaparib monotherapy (Group 4) but was not significantly different from BOLD-100 50 mg/kg monotherapy (Group 3). Furthermore, median tumor growth was delayed for combination of 50 mg/kg BOLD-100 and olaparib (Group 6) compared to the control and all other treatment groups (Figure 7B). Percent group mean body weight did not show any significant changes from Day 1 through Day 24 (Supplement Figure S2). Assessment of gamma-H2AX protein by immunohistochemistry showed increased levels in MDA-MB-231 tumors treated with BOLD-100 (50 mg/kg; Group 3) and combination of 50 mg/kg BOLD-100 and olaparib (Group 6) compared with vehicle (Group 1) or olaparib treated (Group 4) (Figure 8A,B). Collectively, these results showed that BOLD-100 is effective in decreasing TNBC tumor growth in vivo.

## 3. Discussion

BOLD-100 is a novel small molecule that showed modest anti-tumor activity in a Phase I clinical trial [12]. Mechanistically, BOLD-100 has been shown to down-regulate GRP78 in thapsigargin-mediated stressed cancer cells [15] and trigger immunogenic cell death through the PERK/EIF2a branch of the UPR accompanied by ROS production, release of high mobility group box 1 (HMGB1) and ATP secretion via autophagy [31]. Sensitivity toBOLD-100 can vary in different cells and cellular responses including cell cycle (via G2 cell cycle arrest), DNA repair pathway, and cellular metabolism are known to be affected [17]. Here we show that under unstressed conditions, in both ER+ and ER− breast cancer cells, BOLD-100 inhibits cell proliferation. BOLD-100 treatment in ER+ breast cancer cells, significantly altered genes in pathways involved in DNA repair, cell cycle and nucleotide biosynthesis (Figure 3A and Table S1). Mechanistically, we showed that BOLD-100 induces G2/M cells cycle arrest, ROS and gamma-H2AX in both ER+ and ER− breast cancer cells. Furthermore, in ER− breast cancer cells, BOLD-100 combined with olaparib in ER− cells halts autophagy and promotes cell death via apoptosis.

Our gene-metabolite integration model in MCF7 cells offers novel molecular changes associated with BOLD-100 in breast cancer. Downregulation of genes such as ATM or BRCA1 suggest that BOLD-100 treatment leads to the disruption of pathways associated with the DNA repair pathway. Cell cycle proteins such as cyclin D3 (39%) and cyclin E1 (18%) were increased in the RPPA analysis and further work is warranted to determine whether this is a cellular response that contributes to resistance to BOLD-100 with prolonged treatment. Furthermore, levels of gamma-H2AX, a marker of DSB [32], were increased in BOLD-100 treated breast cancer cells (Figure 3C and Figure 6A) and tumors (Figure 8). Monitoring of DSB in human tissues such as blood or skin provides a minimally invasive strategy to monitor efficacy of therapeutics in patients [28], and, therefore, could also be a useful biomarker to evaluate the efficacy of BOLD-100. Furthermore, recent studies have reported changes in cellular metabolite profile [33] or protein associated with specific metabolic pathways [34] including DDR following treatment with BOLD-100. Consistently, our data show an increase in DNA damage in breast cancer cells, as shown by an increase in gamma-H2AX. Specifically, citrate was significantly increased (11-fold) in BOLD-100 treated MCF7 cells (Figure 3D). While further research is needed to define the cellular metabolic changes associated with BOLD-100 treatment in cancer, it is worthwhile to note that citrate is a key metabolite in the tricarboxylic acid cycle (TCA) and increased citrate levels in the cytosol can inhibit growth of cancer cells by G2/M arrest [35].

CYP1B1 gene (Figure 3A) and protein (Figure 3C) was increased with BOLD-100 treatment, which suggests a role of this enzyme in metabolizing the drug. CYP1B1 is a dioxin inducible oxidoreductase and a member of the cytochrome P450 superfamily [36,37]. Single-nucleotide polymorphisms (SNPs) associated with CYP1B1 have been reported to a possible biomarker for therapy response in cancer [21,38,39]. Further studies into the role of CYP1B1 in BOLD-100 mediated inhibition of cancer cell proliferation will be needed to understand whether SNPs associated with this gene can affect drug sensitivity in different cancers.

In TNBC, where there is a lack of targeted therapy options, our data suggests that BOLD-100 alone or in combination with olarapib is effective in inhibiting tumor growth (Figure 7). Olaparib is the only FDA-approved therapy for germline BRCA mutated advanced ovarian cancer and metastatic breast cancer. Olaparib inhibits BRCA1/2 mutated cancer cells that have an increased reliance on PARP to repair their damaged DNA and therefore are more vulnerable to olaparib treatment [8]. Significantly downregulated genes and proteins in MCF7(2) prompted us to test of efficacy of BOLD-100 in combination anticancer therapies that target the DNA in TNBC. Although GRP78 protein levels did not change in MCF7(2) or MDA-MB-231 cells (Figures S1A,B, S7 and S8) following BOLD-100 treatment combination of BOLD-100 and olaparib significantly reduced MDA-MB-231 tumor volume and induced increase in gamma-H2AX compared to control. As reported in the Phase I clinical trial, BOLD-100 has modest anti-cancer effects as a monotherapy [12]. Our in vivo study in human TNBC xenografts confirms the anti-tumor effect of BOLD-100 as a single agent and further demonstrates the benefit of adding BOLD-100 with DNA pathway targeting agents such as olaparib. Currently, BOLD-100 is being tested in combination with other anti-cancer agents for the treatment of various gastrointestinal cancers, including gastric, pancreatic, colorectal and bile duct cancers (NCT04421820). Our study provides a comprehensive view of genes, proteins and metabolites that are changed associated with inhibition of cell growth with BOLD-100 in breast cancer cells. Particularly in TNBC with defective DNA repair pathways, there is potential to use BOLD-100 in effective combination therapeutic approaches.

## 4. Materials and Methods

### 4.1. Cell Culture and Reagents

ER+ MCF7 (originally obtained from the Barbara A. Karmanos Cancer Institute, Detroit, MI, USA), MCF7(2) (a MCF7 derivative cell line that shows increased sensitivity to estrogen; [18] and TNBC cell lines, MDA-MB-231 and MDA-MB-468 (originally obtained from American Type Culture Collection, Manassas, VA, USA), were provided by the Tissue Culture Shared Resources at Georgetown University Medical Center. ER+ breast cancer cells line, MDA-MB-175, was purchased from American Type Culture Collection (ATCC; Manassas, VA, USA). All cell lines were grown in IMEM (Life Technologies, Grand Island, NY, USA; A10489-01) media supplemented with 5% fetal bovine serum. BOLD-100 (formerly IT-139, NKP1339 and KP1339) was provided by Intezyne Biosciences, Inc. (Tampa, FL, USA). For in vitro assays, dimethyl sulfoxide (DMSO) was used at the diluent and negative control (0.2%). Olaparib (AZD-2281) was purchased from Selleck (Houston, TX, USA). All cells were authenticated by DNA fingerprinting and tested regularly for Mycoplasma infection. All other chemicals were purchased from Sigma-Aldrich.

### 4.2. Cell Proliferation and Viability

To determine cell growth, cells were plated in 96-well plates at the following densities: MCF7 and MCF7(2) cells at 4–5 × 10^3^ cells/well. At 24 h, cells were treated with specified drugs for 72 h (or otherwise indicated). For all t = 0 plates, cell media was removed and processed for cell proliferation within 24 h when treatment began. The purpose of the t = 0 plate was to provide a baseline level of cell growth at the beginning of treatment to compare growth with treatment at specified time-points. After treatment, media were removed, and plates were stained with a solution containing 0.5% crystal violet and 25% methanol, rinsed, dried overnight, and re-suspended in citrate buffer (0.1 M sodium citrate in 50% ethanol). Intensity of staining, assessed at 570 nm and quantified using a VMax kinetic microplate reader (Molecular Devices Corp., Menlo Park, CA, USA), is directly proportional to cell number [40,41].

### 4.3. Cell Cycle Analysis

Cells were grown at 70% confluence on 100 mm in complete growth medium for 24 h. The following day, cells were treated with vehicle or 100 μM BOLD-100 for an additional 72 h. Cells were then fixed in ethanol, and analyzed by the Flow Cytometry Shared Resource according to the method of Vindelov et al. [42]. Each experiment was repeated at least three times.

### 4.4. Analysis of Cellular ROS Assay

ROS production was measured by using 2′,7′-dichlorodihydrofluorescein diacetate (DCFDA) as an indicator (ab113851; Abcam, Cambridge, MA, USA). Briefly, cells were plated at 70% confluence on 100 mm dishes in complete media. At 24 h, cells were collected by trypsinization and stained with 10 μM DCFDA dye for 40 min at 37oC. About 1 × 10^5^ cells were aliquoted and treated with the following for 4 h: 100 μM tert-butyl hydrogen peroxide (TBHP; positive control), DMSO alone (negative control), and indicated concentrations of BOLD-100. Fluorescence signal was read at Ex/Em: 485/535 nm through the Flow Cytometry Shared Resource. Changes in ROS levels were determined as a percentage of control after background subtraction.

### 4.5. Western Blotting

Total protein (~20 μg) was isolated from cells following 72 h treatment or vehicle control (0.02% DMSO or ethanol) for protein analysis as previously describe [40,41]. Tris-glycine gels were used for all analysis except for BRCA1, ATM and the respective loading control where Tris-acetate gels were used to accommodate higher molecular weights of these proteins. The following antibodies were used: Actin (#4967), ATM (#2873), BRCA1 (#9010), gamma-H2AX (#80312), Rad51 (#8875), total-H2AX (#7631), p62 (#2947) and LC3BII (#2775) were from Cell Signaling (Danvers, MA, USA); CYP1B1 (ab185954) was from Abcam; β-tubulin (T7816) was from Sigma; DNA/RNA damage antibody (SMC-155) was from StressMarq (Victoria, BC, Canada) and actin (#47778) was from Santa Cruz Biotechnology (Santa Cruz, CA, USA).

### 4.6. Generation, Analysis and Integration of Transcriptomics and Metabolomics Data from MCF7(2) Cells

Transcriptome data: We obtained and analyzed gene expression and untargeted metabolomics data from MCF7(2) cells treated with vehicle alone or 100 μM BOLD-100. Microarray analysis was performed using three biological replicates using Affymetrix HG U133 Plus 2.0 microarray at our Genomics and Epigenomics Shared Resources. Briefly, total RNA was extracted using the RNeasy kit (Qiagen, Valencia, CA, USA). RNA labeling and hybridization were performed according to the Affymetrix protocol for one-cycle target labeling. For each experiment, fragmented cDNA was hybridized in triplicates to Affymetrix GeneChip HG-U95 arrays (Affymetrix, Santa Clara, CA, USA). Affymetrix data analysis included pre-processing of the probe-level Affymetrix data (CEL files). Affymetrix data analysis included pre-processing of the probe-level Affymetrix data (CEL files).

Metabolomics data: Metabolomics analysis was performed through the Metabolomics Shared Resource Core (MSRC) at Georgetown University Medical Center with four biological replicates from each of the two groups, and two technical replicates per sample. LC-MS was used to analyze the MSRC samples. Briefly, metabolite extraction was performed as described by Sheikh et al. [43]. Briefly, the residual pellet was resuspended in 200 μL of solvent A (98% water, 2% ACN and 0.1% formic acid) for Ultra-performance liquid chromatography-electro-spray ionization quadrupole-time-of-flight mass spectrometry (UPLC-ESI-Q-TOFMS) analysis. Mass spectrometry was performed on a Q-TOF Premier (Waters, Milford, MA, USA) operating in either negative-ion (ESI−) or positive-ion (ESI+) electro-spray ionization mode with a capillary voltage of 3200 V and a sampling cone voltage of 20 V in negative mode and 35 V in positive mode. The cone gas flow was 25 L/h, and the source temperature was 120 °C. Accurate mass was maintained by introduction of LockSpray interface of sulfadimethoxine (311.0814 [M+H]+ or 309.0658 [M−H]−). Data were acquired in centroid mode from 50 to 850 m/z in MS scanning. Centroided and integrated mass spectrometry data from the UPLC-TOFMS was processed to generate a multivariate data matrix using MarkerLynx (Waters).

(Accession numbers for all molecular data generated will be available during the review of this manuscript.) Network analysis of the top 2000 differentially expressed (DE) genes, ranked by *p*-value, [IPA Core Analysis (QIAGEN Inc., https://www.qiagenbioinformatics.com/products/ingenuity-pathway-analysis)] identified disease and functional pathways enriched in differentially expressed genes (Table S1). Each of the mutually exclusive networks of DE genes were input into STITCH [19] along with all DE metabolites to integrate the gene and protein expression datasets and expand the networks to include common interacting neighbors from the STITCH database. The resulting networks for each DE set were integrated and pruned of both nodes not detected as differentially expressed in the dataset including subnetworks isolated from the largest interconnected sets. The networks were visualized using Cytoscape [20], with genes represented as rectangles and metabolites as triangles, with the log2 fold change overlaid scaled from low (green) to high (red) expression.

### 4.7. Quantification of Citrate/Isocitrate

Total cellular citrate/isocitrate levels were detected by UPLC-MS in three replicates of MCF7(2) and MDA-MB-231 cells treated with vehicle alone (DMSO) or 100 μM BOLD-100. Briefly, samples were resolved on an Acquity BEH C18 1.7 µm, 2.1 × 100 mm column online with a triple quadrupole mass spectrometer (Xevo-TQ-S, Waters Corporation, Milford, MA, USA) operating in the multiple reaction monitoring (MRM) mode. The sample cone voltage and collision energies were optimized for the analyte to obtain maximum ion intensity for parent and daughter ions using “IntelliStart” feature of MassLynx software (Waters Corporation, Milford, MA, USA). Signal intensities from all MRM Q1/Q3 ion pairs for the analyte were ranked to ensure selection of the most intense precursor and fragment ion pair for MRM-based quantitation. The metabolite ratios were calculated by normalizing the peak area of endogenous metabolites within cell samples normalized to the internal standard (IS-citric acid-d4) and respective levels of proteins from each cell sample. Analysis was performed with a calibration curve, the sample queue was randomized and solvent blanks were injected to assess sample carryover. MRM data were processed using Target Lynx 4.1. The relative quantification values of analytes were determined by calculating the ratio of peak areas of transitions of samples normalized to the peak area of the internal standard.

### 4.8. TNBC Xenografts and In Vivo Studies

In vivo assessment of the efficacy of BOLD-100 and olaparib, alone or in combination, against MDA-MB-231 TNBC xenografts in nice weeks old female NCr nude mice (Crl:NU(NCr)-Foxn1nu, Charles River) through a contract between Intezyne Technologies and Charles River Discovery Services North Carolina. Briefly, cells were implanted subcutaneously and tumors were allowed to form to a mean volume of 107 mm^3^. Bold-100 dosing solution of 5 mg/mL in saline was freshly prepared just prior to administration. Once solubilized in saline, the 5 mg/mL solution was further diluted to 3 mg/mL. The 5 and 3 mg/mL solutions provided the 50 and 30 mg/kg dosages in a dosing volume of 10 mL/kg (200 μL per 20 g animal), scaled to the weight of each animal. Olaparib was freshly prepared weekly by dissolving in one volume of DMSO and further diluting in nine volumes of 20% (2-Hydroxypropyl)-β-cyclodextrin (HPBCD) to achieve a 5 mg/mL dosing solution in 10% DMSO in 20% HPBCD that was administered at 10 mL/kg (50 mg/kg) scaled to the weight of each animal. All dosing solutions were protected from light until dosed. On day 1, mice were divided into six groups (n = 8) and treated as summarized in Table 2. Vehicle control (saline) and BOLD-100 was administered intravenously (i.v.) and olaparib was administered daily orally (p.o.) starting at Day 2 until end of study at Day 28. Tumors were measured twice a week and the study was terminated on Day 28. Tumors were processed for histology. The outcome was determined based on tumor growth inhibition (TGI), which was defined as the difference between the median tumor volumes (MTV) of treated and control mice on day 24, the last day all mice in control Group 1 remained on the study (%TGI = [1 − (MTVdrug-treated/MTVcontrol)] × 100). The results were analyzed utilizing the Mann–Whitney U-test, and were deemed statistically significant at *p* ≤ 0.05. Any treatment that produced at least 60% TGI was considered potentially therapeutically active. Drug tolerability was assessed by body weight measurements (Figure S2) and by frequent observation. These in vivo xenograft studies have been approved by the Animal Care and Use program at Charles River (IACUC ASP #980702 approved December 2017) that is accredited by the Association for Assessment and Accreditation for Laboratory America Care International (AAALAC).

### 4.9. Immunohistochemistry (IHC)

Immunostaining was performed on 5 μm thick sections from MDA-MB-231 xenografts (n = 3) embedded in paraffin with an antibody to gamma-H2AX [phospho S139] (abcam #ab81299) at 1:4000 or a non-specific negative control antibody using the diaminobenzidine (DAB) method at the Histopathology and Tissue Shared Resource. Slides were photographed with bright field using an Olympus IX-71 Inverted microscope at the Microscopy Shared Resource. Semi-quantitative determination of gamma-H2AX IHC images was determined using ImageJ software (National Institute of Health, Bethesda, MD, USA; version 1.53a) [44].

### 4.10. Reverse Phase Protein Array (RPPA)

Whole cell lysates from sub-confluent MCF7(2) cells treated with either 100 mM BOLD-100 or vehicle alone (n = 3 for each condition) for 72 h were analyzed for 305 proteins and phosphoproteins at the RPPA core at the University of Texas MD Anderson Cancer Center [22] following their sample preparation protocol. Briefly, cells were washed in ice-cold PBS, then lysed in RPPA lysis buffer [1% Triton X-100, 50 mM HEPES, pH 7.4, 150 mM NaCl, 1.5 mM MgCl2, 1 mM EGTA, 100 mM NaF, 10 mM Na pyrophosphate, 1 mM Na_3_VO_4_, 10% glycerol, with freshly added protease [#05056489001] and phosphatase [#04906837001] (Roche, Penzberg, Germany) for 30 min on ice, centrifuged for 15 min at 14,000 rpm, and the supernatant collected. Protein concentration was determined using a Pierce 660-nm Protein Assay (ThermoFisher, Waltham, MA, USA). Cell lysates were mixed with sample buffer, boiled and stored at −80 °C until sample submission. Relative quantification of each sample was compared with a reference standard curve generated from control lysates. All data points were normalized for protein loading and transformed to linear value that were used in this study. 

### 4.11. Statistical Analysis and Drug Interaction for Cell Proliferation Experiments

Statistical analyses were performed using the Prism 7 (La Jolla, CA, USA). All experimental values were expressed as mean ± standard errors. Differences between two groups were determined by using the unpaired Student *t*-test, and *p*-values less than 0.05 were considered as statistically significant. The nature of interaction between drugs was defined by measuring the R-index (RI). The RI values were obtained by calculating the expected cell survival (Sexp; the product of survival obtained with drug A alone and the survival obtained with drug B alone) and dividing Sexp by the observed cell survival in the presence of both drugs (Sobs). Sexp/Sobs > 1.0 indicates a synergistic interaction [26].

## 5. Conclusions

BOLD-100 is a novel anticancer drug that has shown a manageable safety profile in a clinical trial. While BOLD-100 is known to decrease the levels of GRP78 in stressed breast cancer cells, the mechanism of action in non-stressed cells remains unknown. Using an unbiased approach, we evaluated the molecular changes associated with the BOLD-100-mediated inhibition of cell growth in breast cancer cells and found DNA repair pathways to be significantly impaired. Therefore, the combination of BOLD-100 with standard anticancer agents that target the DNA, particularly in TNBC, is a reasonable combination strategy. Together, our study contributes to the growing body of work focused on BOLD-100, which shows that the effect of this drug as an anticancer therapy is cell context-dependent. Further studies are warranted to assess whether BOLD-100 could be an effective therapy for metastatic breast cancer.

## Figures and Tables

**Table 1:** Reserve phase protein array (RPPA) results with significantly altered proteins in MCF7 cells treated with 100 μM BOLD-100 compared with control.

PROTEIN NAME	*p*-Value	≥20% Difference in 100 μM BOLD-100 Treated Cells
PDK1	<0.001	24% increased
TSC1	<0.001	27% increased
SOD2	<0.001	22% increased
G6PD	<0.001	39% increased
HER2	0.002	31% increased
PDK1_pS241	0.004	35% increased
Notch1	0.006	21% increased
TFAM	0.006	30% increased
PR (Progesterone receptor)	0.009	64% decreased
DJ1	0.010	24% increased
MUC1 (EMA)	0.014	65% increased
UGT1A	0.015	36% increased
Collagen-VI	0.025	21% increased
c-Jun_pS73	0.026	20% decreased
ATRX	0.031	24% decreased
LC3A-B	0.035	21% increased
Cyclin-D3	0.044	39% increased

**Table 2:** MDA-MB-231 in vivo xenograft study design as of Day 1.

Group	n	Administration of BOLD-100				Administration of Olaparib			
		Drug	Route	mg/kg	Schedule	Drug	Route	mg/kg	Schedule
#1	8	Vehicle	iv	--	qd4 to end	--	--	--	--
#2	8	BOLD-100	iv	30	qd4 to end	--	--	--	--
#3	8	BOLD-100	iv	50	qwk to end	--	--	--	--
#4	8	olaparib	po	50	qd × 32 (start on Day 2)	--	--	--	--
#5	8	BOLD-100	iv	30	q4d to end	olaparib	50	po	qd × 32 (start on Day 2)
#6	8	BOLD-100	iv	50	qwk to end	olaparib	50	po	qd × 32 (start on Day 2)

## Supplementary Materials

The following are available online at https://www.mdpi.com/2072-6694/12/9/2647/s1, Table S1: Top 6 identified disease and functional pathways enriched in differentially expressed (DE) genes in MCF7(2) cells; Table S2: Complete list of Reserve Phase Protein Array (RPPA) results with significantly altered proteins in MCF7 cells treated with 100 μM BOLD-100 compared with control; Figure S1: GRP78 protein levels did not change in basal unstressed breast cancer cells; Figure S2: Mean body weight of mice for in vivo MDA-MB-231 xenograft study; Figure S3: Whole blot for Figure 3B; Figure S4: Whole blot for Figure 3C; Figure S5: Whole blot for Figure 6A; Figure S6: Whole blot for Figure 6B; Figure S7: Whole blot for Supplementary Figure S1A; Figure S8: Whole blot for Supplementary Figure S1B.
