# Supplementary material for: Inhibition of DNA Repair Pathways and Induction of ROS Are Potential Mechanisms of Action of the Small Molecule Inhibitor BOLD-100 in Breast Cancer
Source: Cancers (Basel). 2020 Sep 16;12(9):2647. doi: 10.3390/cancers12092647 (PMC7563761; doi:10.3390/cancers12092647)
Supplement: Supplementary file 1 [file cancers-12-02647-s001.pdf]

Supplementary Materials

Table S1: Top 6 identified disease and functional pathways enriched in differentially expressed (DE) genes in MCF7(2) cells. Significantly changed genes following treatment with 100 μM BOLD-100 were ranked by p-value (IPA Core Analysis, QIAGEN).

# DE GENES	TOP DISEASE AND FUNCTIONS
33	RNA Post-Translational Modification, Cancer, Cell-mediated Immune Response
32	Gene Expression, Connective Tissue Disorders, Developmental Disorders
31	Drug Metabolism, Endocrine System Development and Function, Lipid Metabolism
31	Cancer, Cell Cycle, Cellular Assembly and Organization
31	Embryonic Development, Cell Cycle, Cellular Development
29	Lipid Metabolism, Small Molecule Biochemistry, Vitamin and Mineral Metabolism

Table S2. Complete list of Reserve Phase Protein Array (RPPA) results with significantly altered proteins in MCF7 cells treated with 100 μM BOLD-100 compared with control.

PROTEIN NAME	p-value	% Difference in 100 μM BOLD-100 treated cells
Rab11	<0.001	19% increased
PDK1	<0.001	24% increased
Notch3	<0.001	16% increased
TSC1	<0.001	27% increased
14-3-3-beta	<0.001	12% increased
p38-MAPK	<0.001	16% increased
SOD2	<0.001	22% increased
G6PD	<0.001	39% increased
NAPSIN	<0.001	11% decreased
PI3K-p85	0.001	6% increased
HER2	0.002	31% increased
ACC1	0.002	13% increased
PEA-15	0.002	12% increased
PCNA	0.002	8% decreased
Raptor	0.002	5% decreased
RSK	0.002	11% increased
DM-K9-Histone-H3	0.003	6% decreased
Rad51	0.003	10% decreased
HSP70	0.004	18% increased
PDK1_pS241	0.004	35% increased
Rock-1	0.004	18% increased
CD44	0.004	7% decreased
Jak2	0.004	15% decreased
ATM	0.005	18% decreased
Oct-4	0.005	6% decreased
b-Catenin	0.005	7% decreased
P-Cadherin	0.005	12% increased
Transglutaminase	0.005	5% increased
D-a-Tubulin	0.005	8% increased
Notch1	0.006	21% increased
TFAM	0.006	30% increased
INPP4b	0.007	19% increased
Chk2	0.007	8% decreased
Chk1_pS296	0.007	4% decreased
14-3-3-zeta	0.009	17% increased
PR (Progesterone receptor)	0.009	64% decreased

DJ1	0.010	24% increased
β-Actin	0.010	13% increased
Annexin-VII	0.010	16% increased
p27_pT198	0.011	14% decreased
MAPK_pT202_Y204	0.013	7% decreased
p70-S6K1	0.013	15% increased
ATR_pS428	0.014	12% decreased
TIGAR	0.014	15% increased
MUC1 (EMA)	0.014	65% increased
UGT1A	0.015	36% increased
RBM15	0.015	19% decreased
FRA-1	0.016	7% increased
TUFM	0.017	16% decreased
Bax	0.017	12% increased
Smad4	0.019	4% decreased
HER2_pY1248	0.020	6% increased
MEK1	0.020	7% increased
Stat3	0.021	9% increased
PKCa	0.021	4% increased
MSI2	0.022	10% decreased
RPA32	0.022	5% decreased
p53	0.022	7% increased
AMPK-a2_pS345	0.023	12% decreased
Collagen-VI	0.025	21% increased
c-Jun_pS73	0.026	20% decreased
Smad3	0.026	8% decreased
ATM_pS1981	0.027	3% decreased
TAZ	0.030	13% increased
HER3	0.031	9% increased
ATRX	0.031	24% decreased
Cyclin-E1	0.031	18% increased
Mcl-1	0.031	15% increased
TWIST	0.032	6% decreased
Elk1_pS383	0.032	4% decreased
LC3A-B	0.035	21% increased
IR-b (INSRB)	0.038	9% increased
Rb	0.038	4% decreased
PKC-b-II_pS660	0.039	10% increased
WIPI2	0.042	4% decreased
Ets-1	0.042	7% decreased
Stathmin-1	0.042	3% decreased
Paxillin	0.043	11% increased
IRS1	0.043	16% decreased
Cyclin-D3	0.044	39% increased
ERCC5	0.045	5% increased
p27-Kip-1	0.048	2% decreased

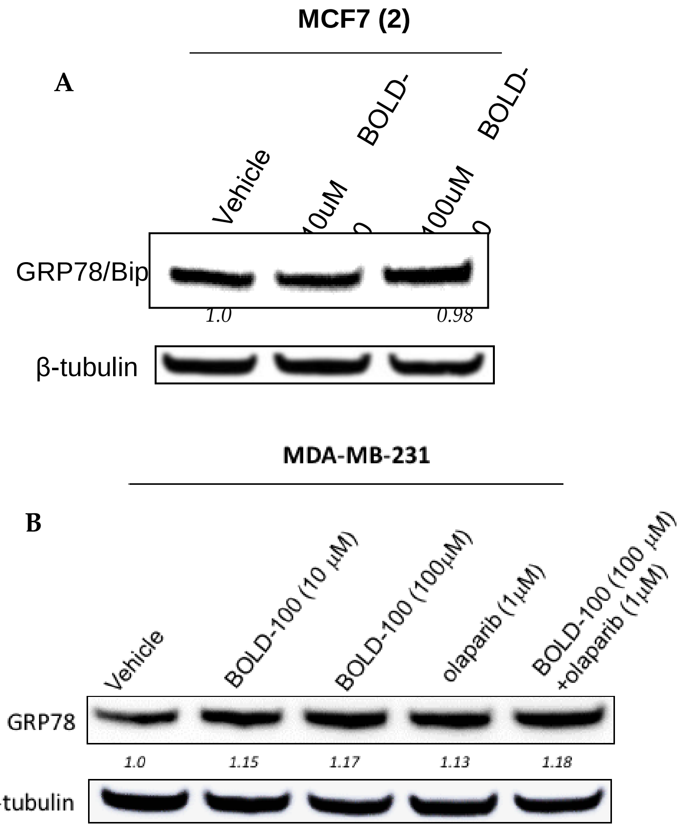

Figure S1. GRP78 protein levels did not change in basal unstressed breast cancer cells cells. (A) Western blot analysis of MCF7(2) cells that were treated with BOLD-100 at indicated doses for 72 h did not show any change in GRP78 levels compared to vehicle Densitometry ratios (protein: loading control) of bands for all conditions versus vehicle control are shown. (B) GRP87 protein levels were determined in MDA-MB-231 cells by Western blotting. Cells were treated with indicted doses of BOLD-100 and/or olaparib for 72 h. β-tubulin was used as the loading control in both cases. For whole blots, please see Figures S7 and S8..

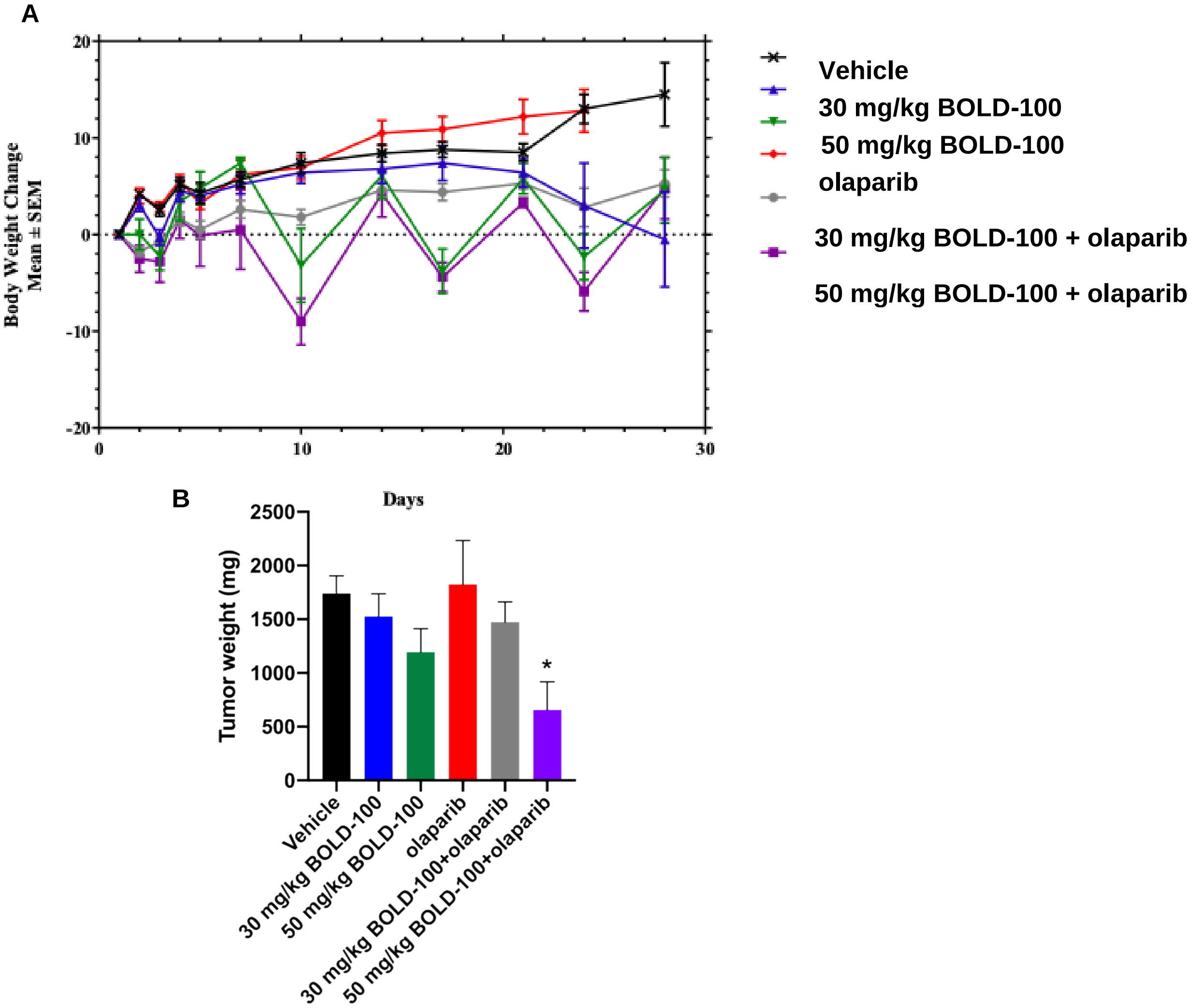

Figure S2. Mean body weight of mice for in vivo MDA-MB-231 xenograft study. (A) Mean body weights were calculated from Day 1 for each group for each day of body weight measurement, and were plotted as a function of time. Error bars on the plots indicate one standard error of the mean (SEM). (B) Tumor weights from each treatment group (n=4) show significantly (*p <0.01) decreased tumor weight in mice treated with BOLD-100 (50 mg/kg) + olaparib compared with that in vehicle treated mice (ANOVA, p <0.05).

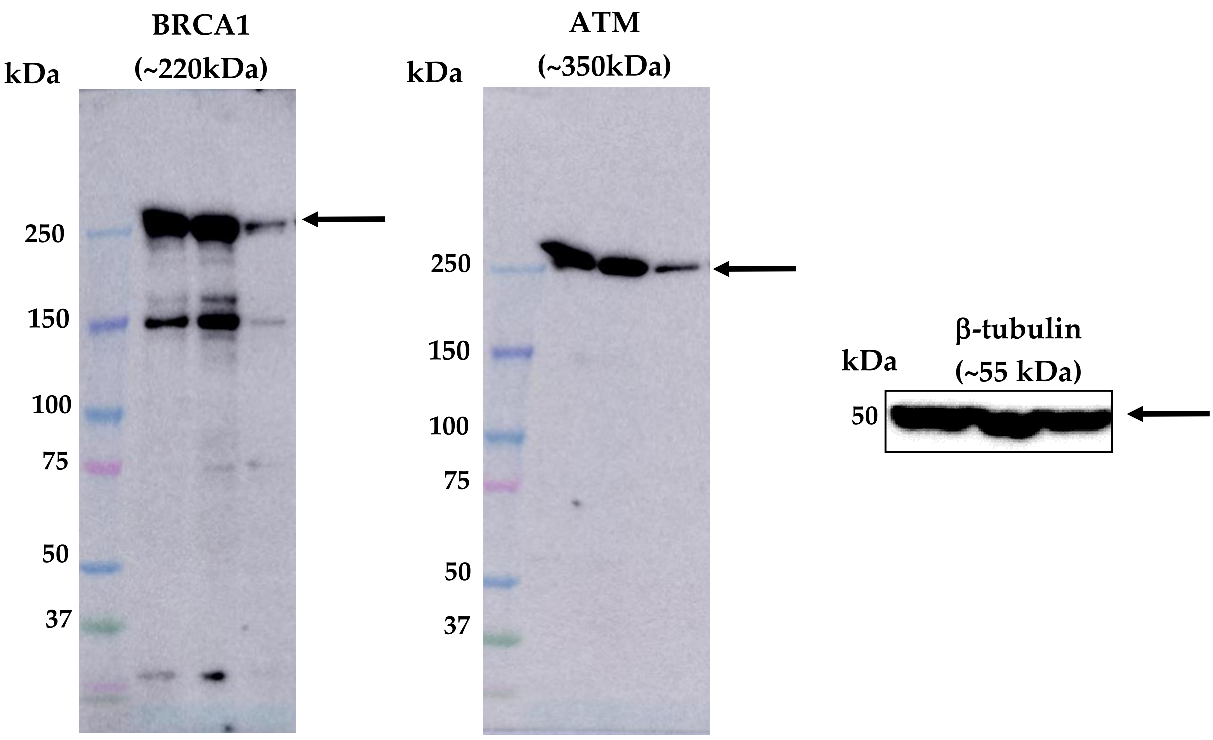

Figure S3. Whole blot for Figure 3B. Note: BRCA1 and ATM we reanalyzed with Tris-acetate gels to accommodate the large size of these proteins. All other gels were Tris-glycine (see Section 4.5). Membranes were stripped and reblotted with different antibodies. For β-tubulin, only cropped image is available since this antibody reliably shows one band and the image of the whole blot was not saved. .

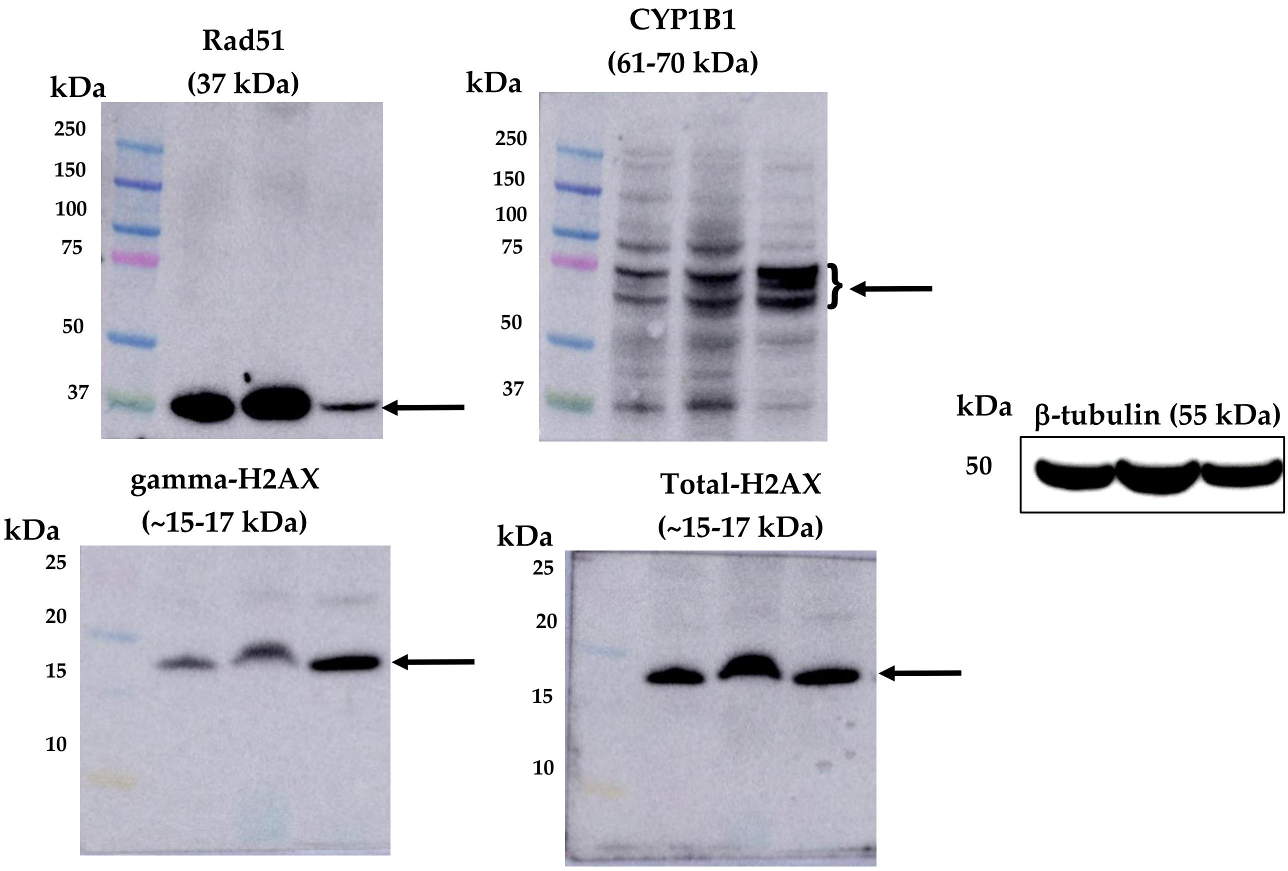

Figure S4. Whole blot for Figure 3 C. Note: This blot was cut and top portion was used to detect RAD51 and then stripped to detect CYP1B1. Bottom portion of blot was used to detect gamma-H2AX and then it was stripped and reused to detect total H2AX. For β-tubulin, which consistently produces one band, only image of the 55 kDa band was saved.

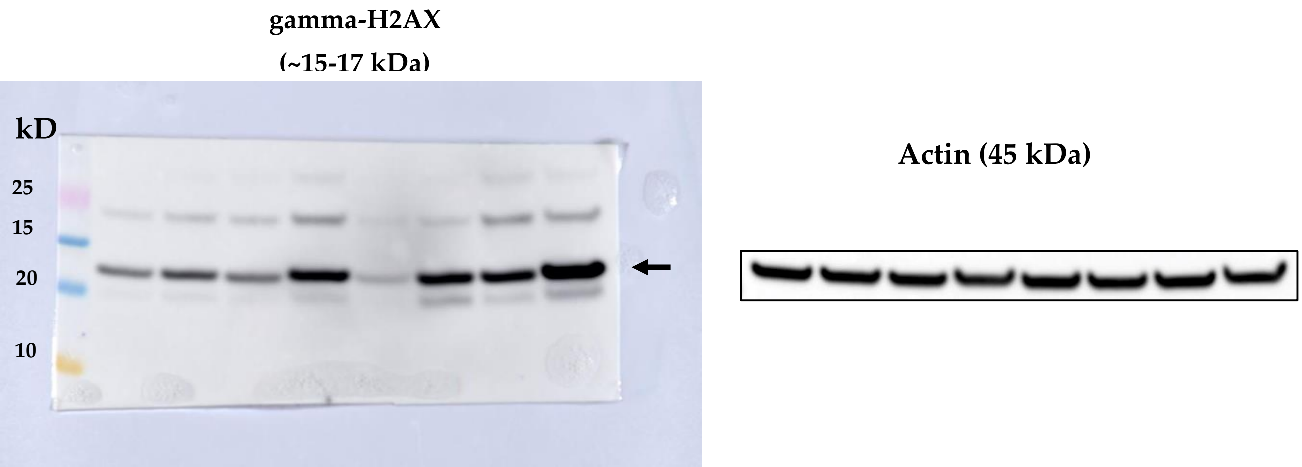

Figure S5. Whole blot for Figure 6 A. Note: This blot was cut and bottom portion was used for gamma-H2AX. For actin, which consistently produces one band, only the 45 kDa band was saved.

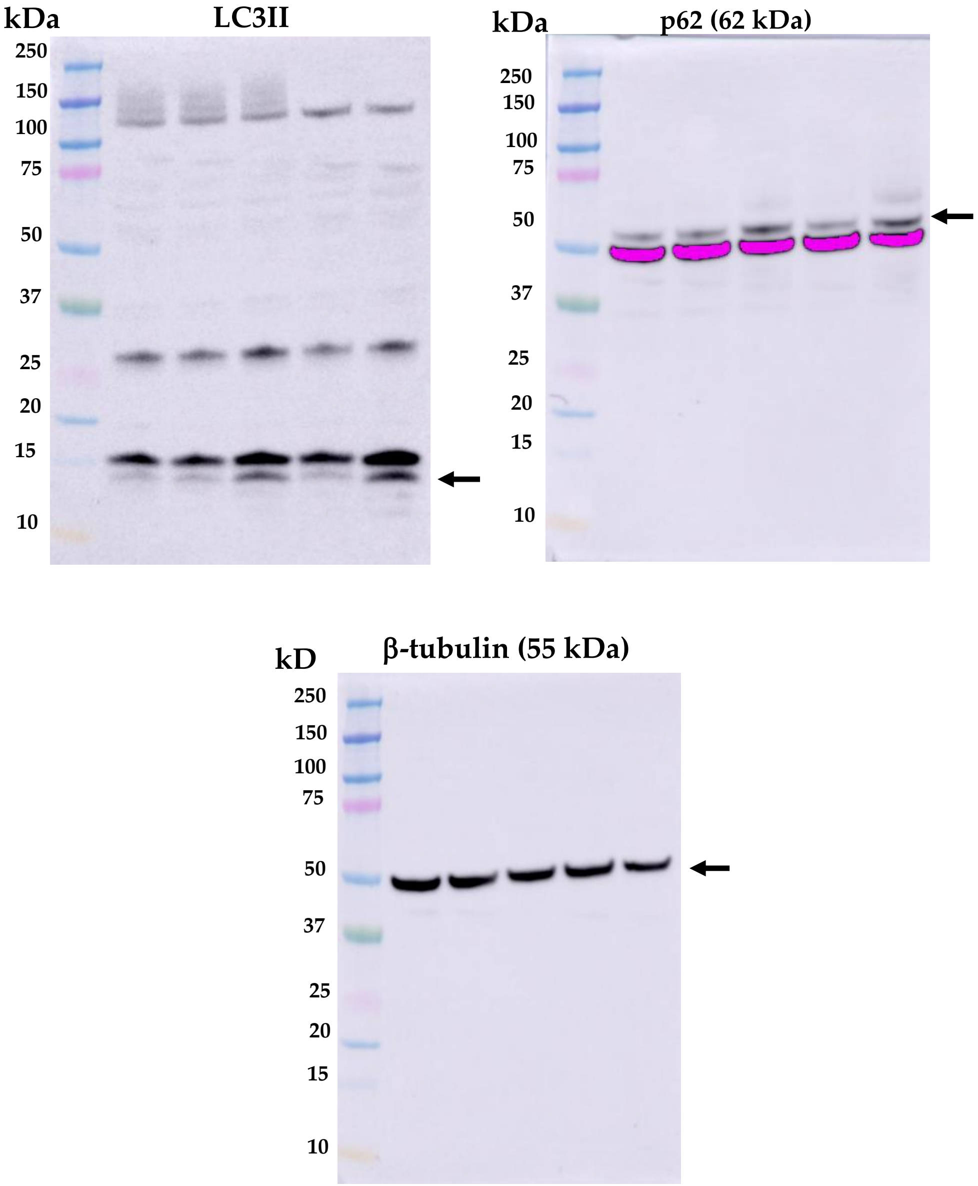

Figure S6. Whole blot for Figure 6 B. Note: This blot used for LC3II and then stripped and blotted for β-tubulin followed by p62.

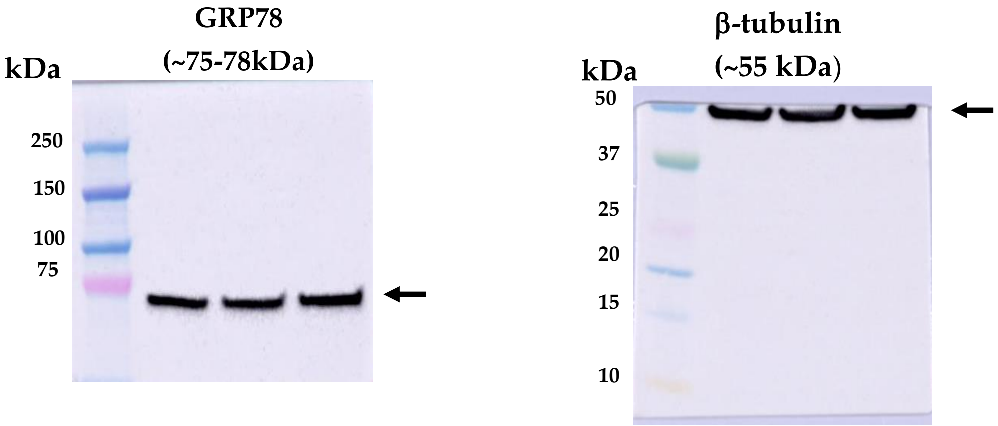

Figure S7. Whole blot for Supplementary Figure S1. Note: This blot was cut and top portion was used for GRP78 and bottom portion was used for β-tubulin.

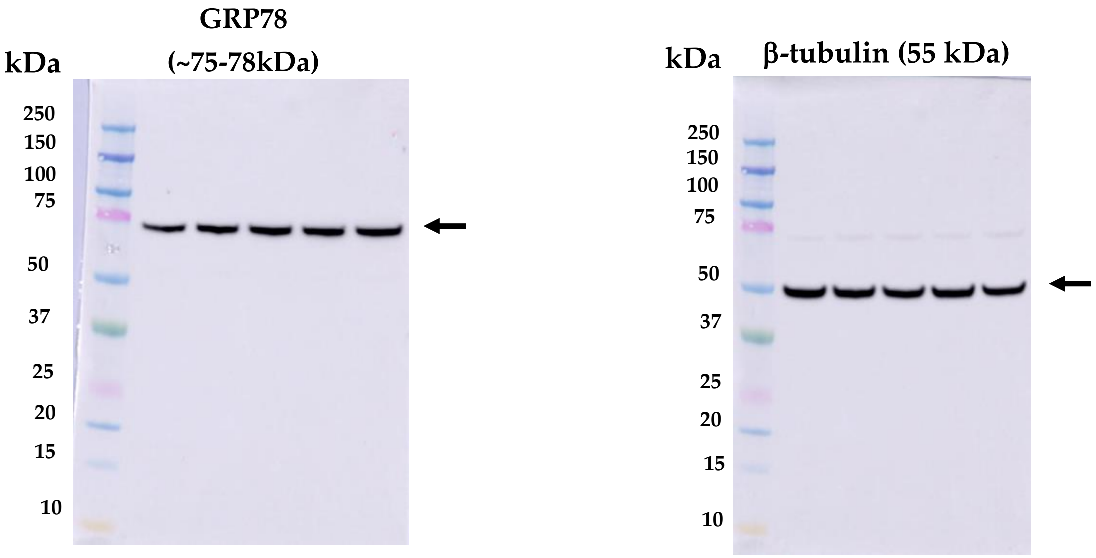

Figure S8. Whole blot for Supplementary Figure S1 B. Note: This blot was first used to detect GRP78, stripped and then used for β-tubulin. .
